# Perceived barriers to accessing physical therapy services in Florida among individuals with low back pain

**DOI:** 10.3389/frhs.2022.1032474

**Published:** 2022-12-08

**Authors:** W.J. Hanney, M.D. Munyon, L.C. Mangum, M.J. Rovito, M.J. Kolber, A.T. Wilson

**Affiliations:** ^1^ Musculoskeletal Laboratory, Institute of Exercise Physiology and Rehabilitation Sciences, School of Kinesiology and Physical Therapy, University of Central Florida, Orlando, FL, United States; ^2^ Orblytics, LLC, Orlando, FL, United States; ^3^ Rehabilitation, Athletic Assessment & Dynamic Imaging (READY) Lab, Institute of Exercise Physiology and Rehabilitation Sciences, School of Kinesiology and Physical Therapy, University of Central Florida, Orlando, FL, United States; ^4^ Department of Health Sciences, University of Central Florida, Orlando, FL, United States; ^5^ Department of Physical Therapy, Nova Southeastern University, Fort Lauderdale, FL, United States

**Keywords:** low back pain, access, healthcare, barriers, conservative care

## Abstract

**Background:**

Low back pain (LBP) affects up to 84% of adults and physical therapy (PT) has been reported to be an effective approach to conservative care. For those individuals with LBP referred to PT, the decision to initiate and follow through with care is influenced by numerous factors. Currently, a paucity of evidence exists to identify barriers for patients with LBP to access PT care. Thus, the purpose of this study was to investigate perceived barriers influencing the decision to pursue PT care in the state of Florida.

**Methods:**

A purposive survey was administered *via* Qualtrics ESOMAR. Screener questions ensured candidates had LBP, resided in Florida, and were referred to PT. Participants that met the screener questions were offered an opportunity to participate in the full survey. Once a participant completed the full survey, variables assessing LBP, access to PT services, and potential barriers were analyzed. A partial least squares structural equation model (PLS-SEM) *via* WarpPLS 7.0 was used to explore which of the perceived barriers had the greatest influence on whether an individual with LBP was able to pursue PT care.

**Results:**

The conceptual framework that demonstrated the best fit of direct effects of potential barriers to accessing care included six independent exogenous latent variables: (a) unaware of a PT clinic near their home or work, (b) had children but no childcare for them, (c) had long PT sessions (e.g., 60 min), (d) had more than one PT session per week, (e) had fewer days active per week, and (f) exercised fewer times per day. Together the six variables explained 19% of the variance related to following through with care (*R*^2^ = 0.19).

**Conclusions:**

The ability of an individual with LBP to access PT care in the state of Florida is multifactorial. There appears to be three broad factors that are the primary barriers, which include (a) the logistic ability (location and access to childcare) to attend PT treatment, (b) how much time is dedicated to the PT treatment, and (c) activity frequency of the individual seeking care. These findings support previous conceptual frameworks for predicting PT treatment. Practitioners and policy makers should consider these barriers when developing plans for conservative management of LBP in Florida.

## Introduction

Low back pain (LBP) is one of the most common musculoskeletal conditions treated in the United States health care system. The point prevalence has been reported to be as high as 29% (1) and the 1-year prevalence rate in the United States ranges from 8% to 56% (2). Estimates suggest between 40% and 85% of people with LBP have sought care from a health care professional (3–5). There are many factors that may influence those suffering with LBP to seek care. For example, patients with acute LBP that initially consulted with a Chiropractor demonstrated an increased willingness to seek care when compared to those who saw an allopathic or osteopathic physician (6). Also, those with recurrent episodes of LBP were more likely to visit the same type of practitioner they had seen for their initial episode as much as 88% of the time (6). Moreover, patients that have worse health overall tend to pursue care more often when compared to those with an improved health status (7). Another strong indicator as to whether a patient will seek care relates to perceived pain intensity and disability (8, 9).

There are numerous treatment options for LBP, which may include surgery or utilization of opioid medications; however, these involve significant risks and side effects (10, 11). One treatment approach that has demonstrated a positive influence on many experiencing LBP, with minimal risks comparatively is treatment by a physical therapist (PT) (12, 13). Interventions delivered by PT practitioners have been found to be efficacious for improving pain, disability, function, as well as fear avoidance beliefs in those individuals with LBP (12, 13).

Recent evidence suggests those with LBP treated by a physical therapist in the early stages of their condition or onset of symptoms are less likely to utilize opioid medications, undergo advance-imaging studies (magnetic resonance imaging (MRI), computed tomography (CT)), or have surgery (12–15). When individuals were treated early by a physical therapist for an episode of LBP, decreased health care utilization and an ultimate decrease in health care costs were observed (16). Essentially, delayed care may lead to a less desirable outcome.

Physicians regularly refer patients for PT services; however, studies report less than 20% of patients with acute LBP actually received PT treatment (12, 13, 17). Failure to follow through with recommended or needed treatment could delay recovery, exacerbate the condition, contribute to further disability, all of which can possibly lead to future increased expenses associated with health care services. Factors related to poor compliance with treatment recommendations include cost, convenience, and perceived value (18–20).

There are many factors that influence access to PT for LBP which creates health equity issues. For example, Medicaid as the health insurance payer has been associated with lower referral rates to PT for persons with LBP (21). Furthermore, out-of-pocket expenditure, physical component score of the SF-12, and the age-insurance category 18–64 years old negatively predict PT utilization for patients with LBP (22). One limitation of the aforementioned data is that it was collected between the time periods of 2007–2010 and only assesses number of visits per episode of care as opposed to barriers to initiating care. Furthermore, economic conditions change, and such information must be determined in an ongoing manner if purposeful efforts to increase access are to be successful.

Authors have examined the ability of individuals suffering from LBP to access different health care providers; however, the literature fails to report barriers to accessing these services (23–25). Reporting access metrics without a clear or tangible understanding of barriers to health care restricts a broader understanding of factors that influence access to care. It has been suggested that individuals with chronic health conditions including chronic LBP have significant barriers to accessing health care when compared to those without chronic conditions (25). However, the totality of evaluating multiple barriers has yet to be reported in the literature. This is important as previous research has not identified actionable barriers that could be addressed to improve access to physical therapy services for those suffering from LBP.

In Canada, socio-demographic, clinical and other factors were associated with perceived access to PT services in those with LBP (26). Although performed in Canada where wait times are a significant barrier to accessing health care, this may not be the case in the United States (26, 27). Also, participants were referred by a physician but not screened until they appeared at a PT clinic for triage, and those with the most significant barriers would likely never appear for triage. Therefore, additional factors that influenced even the possibility of receiving care in a PT setting may have been missed.

Currently, there is a lack of evidence which identifies barriers for patients with LBP in accessing care from a physical therapist. Recent evidence suggests that early access to PT services after the onset of an episode of LBP can have a significant impact on health care costs as well as patient outcomes (12, 13, 17). Although, if barriers to accessing PT services are not clearly identified then many patients that may benefit from PT services might not receive the care they need because the patient was never able to seek the appropriate care. Furthermore, the presentation of acute and chronic LBP pain has very different clinical presentations, treatment, and outcomes. However, it is important to identify barriers to accessing physical therapy services regardless of reported duration of symptoms to evaluate broad implications as it related to access concerns.

Factors currently influencing the decision to proceed with care or opt out of care must be determined if barriers are to be understood fully (22). Thus, the purpose of this study was to identify current factors associated with perceived barriers to PT services in patients with LBP.

## Materials and methods

A purposive survey was distributed *via* cross-sectional design to identify perceived barriers for those individuals with LBP referred to PT from June 2021 to August 2021. Potential barriers, as indicated by Aday and Andersen (26), were categorized into six domains, including: (a) Demographics, (b) Financial, (c) Geographic, (d) Time, (e) Condition, and (f) Previous Care. Survey participants were identified utilizing the Qualtrics survey data excerpt and were limited to the state of Florida. The data collection for this paper was generated using Qualtrics software, Version XM of Qualtrics. Copyright © 2020 Qualtrics. Qualtrics and all other Qualtrics product or service names are registered trademarks or trademarks of Qualtrics, Provo, UT, United States. https://www.qualtrics.com. This study was approved by the University of Central Florida Institutional Review Board (STUDY00002289).

### Participants

The Qualtrics data set was utilized to identify potential candidates to participate. Three screening questions were offered to ensure the desired subgroup was identified to collect data from appropriate participants. The 3 screening questions used to identify the subgroup were:

• Are you a resident of the State of Florida? (yes/no)
  ○ If no, the candidate was excluded from participating in the survey.
  ○ If yes, the candidate was asked the following question.
• Have you had an onset of low back pain in the past year that prompted you to see your physician? (Yes/No)
  ○ If no, the candidate was excluded from participating in the survey.
  ○ If yes, the candidate was asked the following question.
• Did your physician, physician's office, or other health care provider refer you to physical therapy (whether you attended or not)? (Yes/No)
  ○ If no, the candidate was excluded from participating in the survey.
  ○ If yes, the candidate proceeded to the formal survey.

Participants started the formal survey if they answered yes to all screening questions and agreed to participate in the survey after providing informed consent.

G*Power 3 statistical software (28) was used to determine a sufficient sample size for the multiple regression analysis with a power of .95 with an alpha level of .05, using seven variables, and an estimated effect size of .30 (Cohen, 2016). The power analysis results yielded a total sample of at least 120 participants were needed for this study.

### Recruitment and qualtrics data set management

Recruitment and survey data collection was coordinated by Qualtrics (Qualtrics XM, Provo, UT). Qualtrics coordinates partners with over 20 online sample providers supplying a network of diverse, quality respondents to their worldwide client base. To exclude duplication and ensure validity, Qualtrics routinely checked every IP address and used unique and sophisticated digital fingerprinting technology. Using our inclusion and exclusion criteria as well as the Qualtrics' participant past-performance algorithm, Qualtrics' Sample Partners System produced a random selection of respondents that were likely to qualify.

Within Qualtrics, respondents were invited to the survey in multiple ways. Potential respondents were sent an email invitation informing them that the survey was for research purposes only, how long the survey was expected to take, and members could unsubscribe at any time. Respondents could also see the survey if they were likely to qualify for it upon signing into a panel portal based on inclusion and exclusion criteria. To avoid self-selection bias, survey invitations did not include specific details about the contents of the survey and were kept very general. To maintain data quality, Qualtrics replaced respondents who finished in less than half the median survey completion length. This is intended to omit individuals that did not take adequate time to read and answer the questions appropriately. In the case where individuals completed the survey in less than half the median time, the subject's data was omitted and replaced by another participant that completed the survey consistent with other participants. This is a standard protocol implemented by Qualtrics in order to maintain data integrity. Within seven days of survey completion, the investigators could review the results and request responses that needed to be replaced due to other quality issues.

### Study variables and outcome measures

Variables identified as potential barriers to assessing PT were based on the conceptual framework proposed by Aday and Andersen (29). The survey was organized into predisposing factors, enabling factors, and needs sections and consisted of six domains of questions to identify perceived barriers to accessing PT services: (a) Demographics, (b) Financial, (c) Geographic, (d) Time, (e) Condition, and (f) Previous Care. In addition, the investigators included variables based on clinical and professional experience specific to PT care.

Demographic questions included information such as self-reported height, weight (body mass), age, race, gender, smoking status, income categories, marital status, and education level. Financial questions included information such as the insurance provider, deductible, copayments, coinsurance, total out of pocket, and income category. Geographic questions inquired about location or travel distance to a PT clinic, transportation concerns, or cost of travel. Questions about time included concerns about the ability to complete the recommend frequency or duration of treatment. Questions regarding condition included duration of symptoms, distribution of symptoms, pain levels, provoking activities, as well as the Oswestry Disability Index (ODI) (30, 31), Fear Avoidance Beliefs Questionnaire (FABQ) (31, 32), and Numeric Pain Rating Scale (NPRS) (33, 34). Finally, questions about previous care inquired about previous care by a physical therapist for the current condition; or any condition; whether they had been treated by any other health care providers if they had a poor previous experience with PT or poor previous experience with other health care providers.

### Data analysis

Descriptive statistics, including frequency counts, were calculated for all baseline data collected regarding perceived barriers to accessing PT care. SPSS 26 (IBM Corp. Released 2019. IBM SPSS Statistics for Windows, Version 26.0. Armonk, NY: IBM Corp) was used for all descriptive analyses. A partial least squares structural equation modeling (PLS-SEM) was used to examine both the measurement and structural models, as guided by Aday and Anderson (26). Furthermore, PLS-SEM *via* WarpPLS 70 (35). was used to analyze the conceptual framework with an algorithm method analysis.

The analysis explored which latent variable constructs influenced the dependent variable of receiving PT care. The conceptual frameworks consisted of 2 types of models: measurement and structural. Assessment of the measurement model allowed us to determine the reliability and validity of the conceptual framework. The structural model measured the actual effects of latent variable constructs on the dependent variable, receiving PT care.

The following is a list of the unobserved latent variable constructs with their observed formative variables noted in parentheses: (a) demographics (race, age, gender, height, weight), (b) financial (co-pays, deductibles, coinsurance, total out-of-pocket costs), (c) geographic (location of PT provider, patient transportation access), (d) condition (perceived disability, acuity of pain), (e) time (frequency of PT visits, duration of care), and (f) previous care (prior experience with PT, prior experience with other physicians).

## Results

A total of 168 individuals completed the survey. The mean age was 48.4 years and approximately 58% were female. The majority of participants held a bachelor's degree and were Caucasian (Tables 1, 2).

**Table 1:** Subject demographics.


Gender	
Male	70 (42.2%)
Female	96 (57.8%)
Age	
Mean	48.44
Standard Deviation	16.70
Range	65 (18–83)
Education	
Less than high school	4 (2.4%)
High school graduate	51 (30.7%)
Associate's degree (2 year)	29 (17.5%)
Bachelor's degree (4 years)	43 (25.9%)
Master's degree	32 (19.3%)
Research or practice doctorate (e.g., PhD, EdD, ScD, DHS)	4 (2.4%)
Professional doctorate (e.g., DPT, MD)	3 (1.8%)
Race	
American Indian or Alaska Native	1 (0.6%)
Asian	1 (0.6%)
Black or African American	19 (11.4%)
Hispanic or Latino	19 (11.4%)
Multi-Racial	4 (2.4%)
White or Caucasian	122 (73.5%)
Marital Status	
Divorced	20 (12.0%)
Married	94 (56.6%)
Single	52 (31.3%)
Smoker	
No	115 (69.3%)
Yes	51 (30.7%)
Activity Level	
Sedentary	39 (23.5%)
Mostly sedentary	23 (13.9%)
Moderately active	95 (57.2%)
Demanding physical activity	9 (5.4%)
Active Days per Week	
1	16 (9.6%)
2	29 (17.5%)
3	34 (20.5%)
4	32 (19.3%)
5	23 (13.9%)
6	9 (5.4%)
7	23 (13.9%)
Exercise Sessions per Day	
0	12 (7.2%)
1	108 (65.1%)
2	29 (17.5%)
3	8 (4.8%)
4 or more	9 (5.4%)
Minutes per Exercise Session	
0–15 min	30 (18.1%)
16–30 min	52 (31.3%)
31–45 min	45 (27.1%)
46–60 min	22 (13.3%)
More than 60 min	17 (10.2%)
Health Insurance: Type	
HMO	32 (19.3%)
PPO	29 (17.5%)
Medicaid	16 (9.6%)
Medicare	61 (36.7%)
Self-Pay	28 (16.9%)
Health Insurance: Co-Pay	
$0–$10	35 (21.1%)
$11–$20	14 (8.4%)
$21–$30	36 (21.7%)
$31–$40	24 (14.5%)
$41–$50	12 (7.2%)
More than $50	16 (9.6%)
I do not know	29 (17.5%)
Health Insurance: Annual Deductible	
$0–$1,000	23 (31.9%)
$1,001–$2,000	18 (10.8%)
$2,001–$3,000	29 (17.5%)
$3,001–$4,000	12 (7.2%)
$4,001–$5,000	5 (3.0%)
More than $5,000	9 (5.4%)
I do not know	40 (24.1%)
Annual Household Income	
Less than $10,000	6 (3.6%)
$10,000–$19,999	10 (6.0%)
$20,000–$29,999	22 (13.3%)
$30,000–$39,999	14 (8.4%)
$40,000–$49,999	13 (7.8%
$50,000–$59,999	20 (12.0%)
$60,000–$69,999	10 (6.0%)
$70,000–$79,999	13 (7.8%)
$80,000–$89,999	6 (3.6%)
$90,000–$99,000	8 (4.8%)
$100,000–$149,000	30 (18.1%)
More than $150,000	14 (8.4%)

**Table 2:** Pain.

Current level of lower back pain while completing survey (0–10)
0	17 (10.2%)
1	7 (4.2%)
2	12 (7.2%)
3	13 (7.8%)
4	15 (9.0%)
5	30 (18.1%)
6	23 (13.9%)
7	23 (13.9%)
8	14 (8.4%)
9	5 (3.0%)
10	7 (4.2%)
Lowest level of lower back pain in past year (0–10)	
0	21 (12.7%)
1	14 (8.4%)
2	24 (14.5%)
3	19 (11.4%)
4	19 (11.4%)
5	19 (11.4%)
6	15 (9.0%)
7	12 (7.2%)
8	10 (6.0%)
9	5 (3.0%)
10	8 (4.8%)
Highest level of lower back pain in past year (0–10)	
0	2 (1.2%)
1	1 (0.6%)
2	1 (0.6%)
3	6 (3.6%)
4	4 (2.4%)
5	9 (5.4%)
6	19 (11.4%)
7	32 (19.3%)
8	26 (15.7%)
9	30 (18.1%)
10	36 (21.7%)

### Financial factors

The type of insurance participants reported was evaluated, which included health maintenance organization (HMO) (19.3%), preferred provider organization (PPO) (17.5%), Self-Pay (16.8%), Medicare (37.4%), and Medicaid (9.0%). Copayments for PT services were evaluated and ranged between 0 to >$50, which included $0–$10 (21.1%), $11–$20 (8.43%), $21–$30 (21.7%), $31–$40 (14.5%), $41–$50 (7.2%), >$50 (9.6%), and “I don't know” (17.5%). In the current study, reported annual deductibles were: $0–$1,000 (31.9%), $1,001–$2,000 (10.8%), $2,001–$3,000 (17.5%), $3,001–$4,000 (7.8%), $4,001–$5,000 (2.4%), >$5,000 (5.4%), and “I don't know” (24.1%). Finally, respondents to our survey reported household incomes to be $30,000 to $39,000 (8.4%), $40,000–$49,000 (7.8%), $50,000–$59,000 (12.1%), $60,000–$69,000 (6.0%), $70,000–$79,000 (7.8%), $80,000–$89,000 (3.6%), $90,000–$99,000 4.8%, $100,000–$149,000 (18.1%), and $150,000 or more (8.4%).

### Previous care

We inquired about a series of factors that may influence access to care based on previous care, and 83.7% had received care for their LBP in the past. Of those that received care, 52.4% saw a physical therapist, 21.1% saw an orthopedic surgeon, 31.3% saw a chiropractor, 38.6% saw a primary care doctor, and 13.2% saw a neurosurgeon. While all participants in the current study were referred to PT for their most recent episode of LBP 75.3% followed through with PT care and 69.3% of those that attended PT care completed the prescribed number of treatment sessions (Table 3).

**Table 3:** Previous care.

Received Previous Care for Lower Back Pain	
Yes	139 (83.7%)
No	27 (16.3%)
Number of Sessions Originally Prescribed	
1–10	69 (41.6%)
11–20	40 (24.1%)
21–30	7 (4.2%)
More than 30	6 (3.6%)
No Response	44 (25.5%)
Follow Through with Previous Care	
Yes	125 (75.3%)
No	14 (8.4%)
Previous Care was Helpful	
Yes	108 (65.1%)
No	27 (16.3%)

### Time requirements

When inquiring about the time necessary to complete each individual PT treatment session, approximately 63% agreed or strongly agreed this would be a potential barrier to accessing PT care. When asking about frequency of treatment each week, approximately 54% indicated three times per week or more would be a barrier to accessing care. Other factors that may influence an individual's ability to participate in PT care include childcare during treatment (33.7%), work schedule, (42.2%), and caring for other family members (33.1%) (Table 4).

**Table 4:** Perceived challenges and barriers to physical therapy.

Awareness of a physical therapy clinic near your home or work	
Yes	146 (88.0%)
No	20 (12.0%)
Approximate distance of the nearest physical therapy clinic	
0–5 miles	50 (30.1%)
6–10 miles	69 (41.6%)
11–15 miles	29 (17.5%)
16–20 miles	14 (8.4%)
More than 20 miles	4 (2.4%)
Challenges	
Personal transportation	
Yes	53 (31.9%)
No	113 (68.1%)
Public transportation	
Yes	71 (42.8%)
No	95 (57.2%)
Road tolls	
Yes	48 (28.9%)
No	118 (71.1%)
Distance of physical therapy center from home and work	
Yes	47 (28.3%)
No	119 (71.7%)
Barriers	
Length of each session	
Less than 30 min	12 (7.2%)
30 min	31 (18.7%)
45 min	33 (19.9%)
60 min	22 (13.3%)
More than 60 min	39 (23.5%)
Not a barrier	29 (17.5%)
Frequency of days per week	
Weekly	25 (15.1%)
Two times per week	22 (13.3%
Three times per week	48 (28.8%)
Daily	42 (25.3%)
Not a barrier	29 (17.4%)
Duration of treatment plan	
1–3 months	44 (26.5%)
4–6 months	46 (27.7%)
7–12 months	25 (15.1%)
More than 12 months	15 (9.0%)
Not a barrier	36 (21.7%)
Children but no childcare	
Yes	56 (33.7%)
No	110 (66.3%)
Care for family, no time for own care	
Yes	55 (33.1%)
No	111 (66.9%)
Work schedule makes it difficult to attend	
Yes	70 (42.2%)
No	96 (57.8%)

### Influential factors on barriers to physical therapy care

First for the PLS-SEM analysis, we examined the reliability and validity indices of the outer model for the corresponding formative and single item constructs (Table 5). The outer weights for the formative constructs seemed adequate. The outer weight for length of treatment more than 60 min (*BarrLong)*, treatment sessions frequency of three times per week (*BarrFreq)*, and time spent in exercise activity 15–30 min were negative (*ActTime)*. The variance inflated factor (VIF) was used to measure the multicollinearity of indicators. All VIF values were well under the recommended value of 5.0 except for *PTCdist*. While *PTCdist* had significantly affected *CareFT*, its VIF values were over 5.0. As a result, we attempted to keep *PTCdist* in the analysis by recoding it as a dichotomous variable (1 = 0–5 miles, 2 = 6–10; the most populous subgroups of the variable). This resulted in a non-significant effect. We then recoded *PTCdist* as a 3-part formative variable (1 = 0–5 miles, 2 = 6–10 miles, and 3 = 11 or more miles). This new formative variable also had no effect on *CareFT* (PT care follow through defined as completed physical therapist's prescribed plan of care). As such, we deleted *PTCdist* from the framework. Despite its deletion from the framework, we were interested in exploring if the variance from the structural model was different among the three sub-samples of *PTCdist* (0–5, 6–10, 11+).

**Table 5:** Assessment of measurement.

Constructs/Items	Type	Loadings/Weights	AVE	CR	VIF
ptcaware	Single Item	N/A	N/A	N/A	0.000
Yes					
No					
chldcare	Single Item	N/A	N/A	N/A	0.000
Yes					
No					
pttime	Formative		N/A	N/A	
Less than 30 min		0.284			1.683
30 min		0.385			1.713
45 min		0.094			1.526
60 min		0.042			1.312
More than 60 min		−0.734			1.794
Ptfreq	Formative		N/A	N/A	
Daily		0.538			2.002
Twice per week		0.098			1.783
Three times per week		−0.652			2.076
Weekly		0.060			1.631
Acttime	Formative		N/A	N/A	
One time per day		0.521			3.494
Two or more times		−0.521			3.494
Actdays	Single Item	N/A	N/A	N/A	0.000

Ptcaware – Physical therapy center, awareness of a center near home; chldcare, child care; pttime, time to attend treatment; ptfreq, frequency of physical therapy sessions; Acttime – activity, average minutes spent per activity.

### Assessment of the structural model

After testing multiple models, the conceptual framework that demonstrated the best fit of direct effects on the dependent endogenous latent variable *careft* included 6 independent exogenous latent variables: (a) *ptcaware* (PT center: awareness of a center near home), (b) *barrcare* (perceived barrier: having children but no childcare for them), (c) *barrfreq* (perceived barrier: frequency of PT sessions), (d) *barrlength* (perceived barrier: length of each PT session), (e) *actdays* [activity: number of days per week with physical activity (exercise)], and (f) *acttime* (activity: average minutes spent per activity). Together the 6 exogenous latent variables explained 18% of the variance related to following through with care (*R*^2^ = 0.18). A significant direct effect was found between the following latent variables and *CareFT*: (a) *PTCaware* (0.20, *p* = <0.01), (b) *barrcare* (0.15, *p* = 0.03), (c) *barrlong* (0.26, *p* = <0.01), (d) *barrfreq* (−0.23, *p* = <0.01), (e) *actdays* (0.19, *p* = <0.01), and (f) *acttime* (0.15, *p* = 0.02) (Table 6).

**Table 6:** Path coefficients.

Relationship	Path coefficient	*t*	95% Bias Corrected CI	*p*	Effect Size (*F*^2^)	Supported
ptcaware → careft	0.212	2.879	[0.068–0.357]	0.002	0.040	Yes
ptfreq → careft	−0.222	−3.017	[−0.366 – −0.078]	0.001	0.047	Yes
pttime → careft	0.247	3.371	[0.103–0.391]	< 0.001	0.049	Yes
actdays → careft	0.172	2.310	[0.026–0.318]	0.011	0.015	Yes
acttime → careft	0.152	2.030	[0.005–0.298]	0.022	0.016	Yes
chldcare → careft	0.149	1.990	[0.002–0.295]	0.024	0.013	Yes

Ptcaware – Physical therapy center, awareness of a center near home; chldcare, child care; pttime, time to attend treatment; ptfreq, frequency of physical therapy sessions; Acttime – activity, average minutes spent per activity; Careft, dependent endogenous latent variable.

## Discussion

This study sought to identify potential barriers to following through with prescribed PT care. The results showed that six latent variables significantly influenced patients' follow through with care. Patients were less likely to follow through with care when they were: (a) unaware of a PT clinic near their home or work, (b) had children but no childcare for them, (c) had long PT sessions (e.g., 60 min), (d) had more than one PT session per week, (e) had fewer days active days per week, and (f) exercised fewer times per day.

The ability to access health care can limited by a multitude of factors and one barrier is the ability to be physically present for treatment. Therefore, the location of a given treatment facility is an important factor and seems to play a crucial role as to whether an individual will be able to participate in care. This is particularly challenging for PT treatments as they often require multiple treatment sessions each week and can span the course of several weeks. Excessive distance to a PT center for treatment can be time consuming and require additional expense for travel related costs.

Individuals have many responsibilities and those required to care for a child or perhaps and elderly parent can plan a significant role limiting access to PT care. Most health care facilities do not offer childcare and then, it is incumbent upon the patient to make arrangement. This can cause the patient to incur additional costs which can add to the overall cost of care. Therefore, patient-related factors such as socioeconomic status may influence compliance with recommended care (23). Again, PT treatments often require multiple treatment sessions over a period of time and therefore the cumulative expense can be an additional barrier to care.

Our results indicated that 75.3% of respondents did follow through with previous care. Therefore, the question must be asked as to whether there was actually an access issue. Three quarters of participants had some type of care which indicates a significant number of respondents were able to access some type of healthcare. While this may be the case, nearly a quarter of respondents did not follow through with care or did not have access to care. Barrier to accessing care in these individuals is important to better understand why some may or may not follow through with prescribed care (36).

As stated, PT treatments sessions are different than many other forms of health care. Active participation by the patient is necessary and treatment must be adapted and progressed in order for physiological adaptations (37–39). This requires ongoing participation of the patient which requires dedicated compliance with prescribed treatment. Ultimately, the number of treatment sessions per week as well as the anticipated number of weeks required to sustain adequate progress in care can be prohibitive as this may create an undue burden secondary to lost wages while missing work to attend treatment, copayments, cost of child support, etc. (36).

An additional finding of interest is the role that an individual's activity level and frequency of exercise played on perceived to accessing PT services (36) It appears that individuals that are less active and participate in exercise less frequently are less likely to attend PT sessions (39, 40). While there are several factors that may influence this finding it may be due to the requirement to be actively involved in PT treatment. Those that may not feel comfortable with actively participating in care may be less likely to attend PT treatment sessions.

The intent of the proposed study was to evaluate barriers to accessing physical therapy services for those with LBP in the state of Florida. The screening questions identified individuals that reported an episode of LBP in the past year; however, we did not collect data regarding the duration of reported symptoms. It should be noted that there is a difference in presentation, treatment and recovery when evaluating chronic vs. acute LBP. However, the primary purpose of the present study was not to evaluate presentation, treatment or recovery and simply perceived barriers to accessing to care. While this would have been an interesting variable to evaluate, the authors only collected the overarching presence of reported symptoms in the past year and any challenges in accessing care. This invites further inquiry in to evaluating barrier to accessing physical therapy services for those with acute vs. chronic LBP.

The current study is not without limitations. Given the time frame in which data collection occurred, the question of COVID influence is important to address. In the state of Florida, COVID restrictions were lifted September 25, 2020. While there may have been some residual impact on access to healthcare, we hope that administering the survey approximately 9 months after conclusion of the Florida shut down would have minimal effect on the results. Unfortunately, we did not collect a variable revaluating the impact of COVID and therefore this would be speculation. Furthermore, this was an exploratory study and therefore we did not cross-validate outcomes in a separate sample. Also, we did not evaluate perceptions associated with these variables which may limit a broader understanding of how participants interpreted the reported variables. In addition, we did not retain distance as a variable and therefore are unable to indicate a specific distance from a PT clinic that would be a significant barrier. Finally, the cross-section nature of the data limits the ability to establish a causal relationship.

In conclusion, LBP has a societal impact and carries a significant economic burden. This is the first study to identify perceived barriers to accessing PT services in the state of Florida which offers unique insight into factors that may play an important role in evaluating how LBP is addressed in this state. Also, a wide range of individuals seek care for LBP, and physicians refer to PT clinics for treatment regularly. PT seems to be an effective treatment option for those suffering with LBP which has fewer potential side effects when compared to pharmacological or surgical interventions, potentially providing significant overall cost savings. It should be noted that this study reports perceived barriers and a significant number of respondents did attend physical therapy treatment.

## Data Availability

The raw data supporting the conclusions of this article will be made available by the authors, without undue reservation.
